# Quantification of ^18^FDG in the Normal Colon—A First Step in Investigating Whether Its Presence Is a Marker of a Physiological Process

**DOI:** 10.1371/journal.pone.0147838

**Published:** 2016-01-28

**Authors:** Karna D. Bardhan, James Cullis, Nigel R. Williams, Ramesh P. Arasaradnam, Adrian J. Wilson

**Affiliations:** 1 Department of Gastroenterology, Rotherham General Hospital, Rotherham S60 2UD, United Kingdom; 2 School of Medicine, University of Sheffield, Sheffield S10 2TN, United Kingdom; 3 Nuclear Medicine, Department of Clinical Physics and Bioengineering, University Hospital, Coventry CV2 2DX, United Kingdom; 4 Department of Gastroenterology, University Hospital, Coventry CV2 2DX, United Kingdom; 5 Clinical Sciences Research Institute, University of Warwick, Coventry CV4 7AL, United Kingdom; 6 Department of Research, Development and Innovation, University Hospital, Coventry CV2 2DX, United Kingdom; 7 Department of Physics, University of Warwick, Coventry CV4 7AL, United Kingdom; Banner Alzheimer's Institute, UNITED STATES

## Abstract

The visibility of the colon in positron emission tomography (PET) scans of patients without gastrointestinal disease indicating the presence of ^18^F Fluorodeoxyglucose (^18^FDG) is well recognised, but unquantified and unexplained. In this paper a qualitative scoring system was applied to PET scans from 30 randomly selected patients without gastrointestinal disease to detect the presence of ^18^FDG in 4 different sections of the colon and then both the total pixel value and the pixel value per unit length of each section of the colon were determined to quantify the amount of ^18^FDG from a randomly selected subset of 10 of these patients. Analysis of the qualitative scores using a non-parametric ANOVA showed that all sections of the colon contained ^18^FDG but there were differences in the amount of ^18^FDG present between sections (p<0.05). Wilcoxon matched-pair signed-rank tests between pairs of segments showed statistically significant differences between all pairs (p<0.05) with the exception of the caecum and ascending colon and the descending colon. The same non-parametric statistical analysis of the quantitative measures showed no difference in the total amount of ^18^FDG between sections (p>0.05), but a difference in the amount/unit length between sections (p<0.01) with only the caecum and ascending colon and the descending colon having a statistically significant difference (p<0.05). These results are consistent since the eye is drawn to focal localisation of the ^18^FDG when qualitatively scoring the scans. The presence of ^18^FDG in the colon is counterintuitive since it must be passing from the blood to the lumen through the colonic wall. There is no active mechanism to achieve this and therefore we hypothesise that the transport is a passive process driven by the concentration gradient of ^18^FDG across the colonic wall. This hypothesis is consistent with the results obtained from the qualitative and quantitative measures analysed.

## Introduction

The presence of ^18^F Fluorodeoxyglucose (^18^FDG) in the colon of patients undergoing a positron emission tomography (PET) scan to investigate non-gastrointestinal malignancies (for example, liver and lung metastases) is widely recognised [1,2] but its clinical and physiological significance has yet to be explained.

^18^FDG is a deoxyglucose which, when injected intravenously, follows the glucose transport pathways but, importantly, is not metabolised and hence accumulates in cells in proportion to their metabolic rate. Some tumour cells take up more ^18^FDG than normal cells because of their higher metabolic rate creating regions of ‘higher activity’ on the resultant PET images. This differential uptake of ^18^FDG is the principle underlying the diagnostic value of PET scans in clinical practice [1].

Being a glucose-based molecule, the presence of ^18^FDG in the colonic lumen seemed counter-intuitive to gastroenterologists, as any glucose in the fore- and mid-gut is very rapidly absorbed and hence none should reach the colon. The anecdotal evidence from radiologists is that ^18^FDG localisation in the colon occurs frequently in patients without gastrointestinal disease. However, they attach no clinical significance to the presence of ^18^FDG in the colon generally viewing it as artefact, ‘noise’ or ‘normal physiological uptake’.

Determining the precise location of ^18^FDG within the colon, whether it is in the colonic wall, the lumen or both, is difficult because of the limited spatial resolution of PET scanners (typically 4mm). In comparison, the colon is a tubular structure approximately 150cm in length, its diameter varying from 2.5 cm to 7.5 cm respectively in the sigmoid and caecum [3] with a wall thickness of 10–20 mm, varying by location, colonic diameter, and the method of measurement [4–8].

These observations directly led us to question how intravenous ^18^FDG was transported to the colon. Based on known anatomy and physiology, two options seemed possible. The first is through its secretion in the bile. The second, which to us seems more likely, is that the ^18^FDG is driven through the colonic wall by its blood-to-lumen concentration gradient, a passive process and a mechanism which hitherto has not been considered.

Despite the frequency of ^18^FDG localisation in the colon, we could not find a report giving a good estimate of incidence with some studies reporting an absence of ^18^FDG in the colon of some subjects [9,10] whilst another study reports subjects with an absence of ^18^FDG in one or more segments of the colon [11]. Therefore in this pilot study, we sought to determine: (a) how frequently ^18^FDG activity is found within different segments of the colon; (b) to quantify that activity; and finally (c) consider mechanisms which might explain the presence of ^18^FDG in the colon.

## Methods

Ethical consideration (UHCW Trust Research & Development office) was considered unnecessary as only digital anonymised images were reviewed with no patient identifiable material. The authors had no direct patient contact and case notes were not reviewed hence no indication to obtain patient consent. All prior information had already been de-identified prior to analysis by an independent personnel.

**Fig 1:**
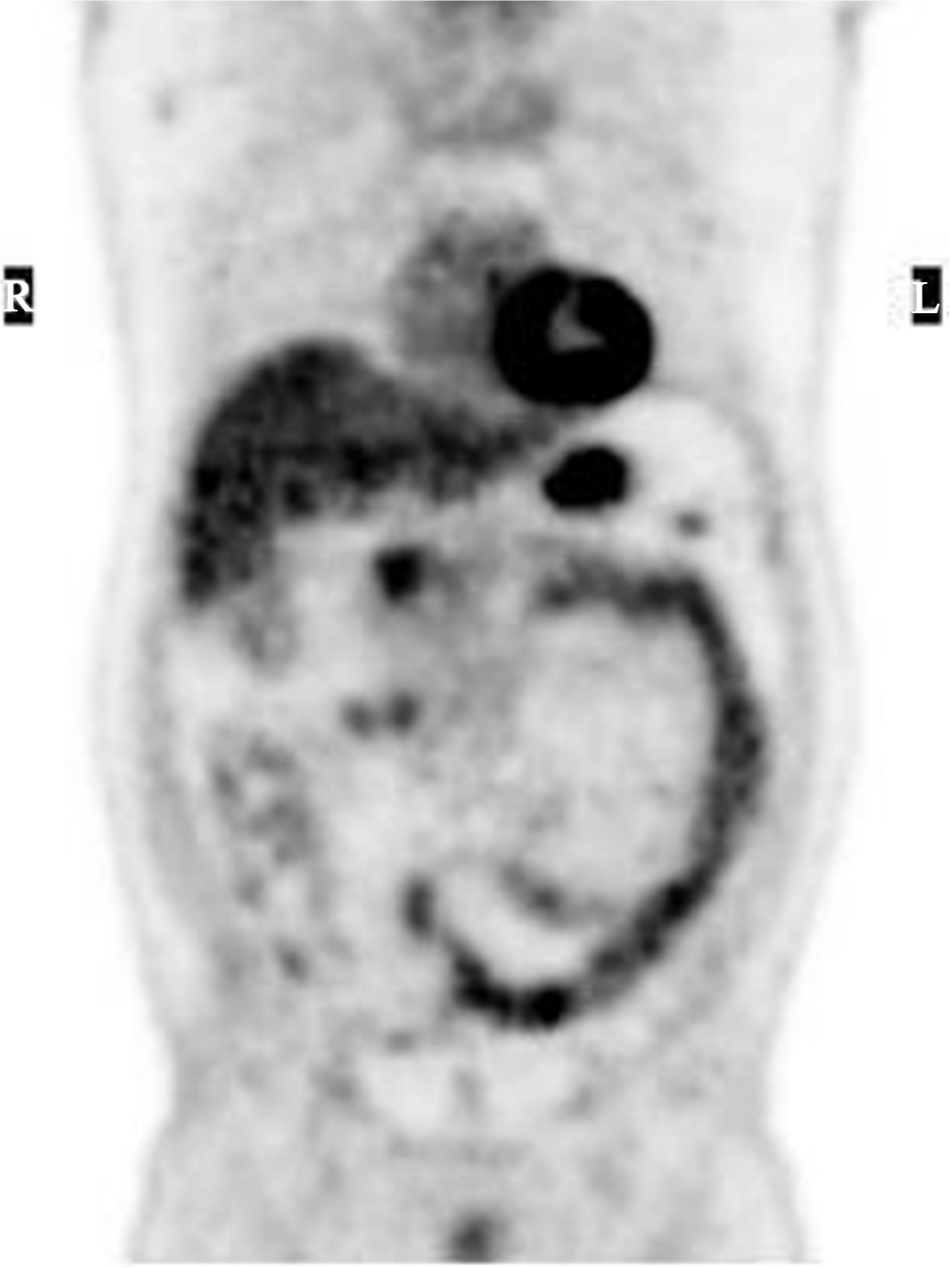
Image clearly showing ^18^FDG the in the descending colon (DC) and the sigmoid and rectum (S&R). The outline of the caecum and ascending colon (C&AC) can also be clearly seen. Note that only the left hand end (right hand end on the image) of the transverse colon (TC) is clearly visible. The transverse colon (TC) is approximately horizontal and extends from just below the lowest point in the liver (which can be clearly seen) to the top of the descending colon.

The study population consisted of 30 anonymised scans from different patients who underwent routine PET/CT imaging to investigate suspected non-gastrointestinal malignancies (principally head and neck cancers). Three patients had type 11 Diabetes mellitus but none were on Metformin therapy. Following intravenous injection of ^18^FDG the patients rested for 60 minutes before being imaged on a GE Discovery STE scanner. The acquired PET/CT data were reconstructed in a routine manner (iterative reconstruction, two iterations and 20 subsets) leading to the creation of transaxial, sagittal and coronal image sets. Five observers familiar with this form of imaging were asked to score the amount of activity in each of four colonic segments using a visual analogue scale. Zero indicated no activity and a value 1–5 indicated different levels of activity, where 5 was allocated to the segment with the highest activity for a given patient. The four segments of the colon were: ‘caecum & ascending colon’ (C&AC), ‘transverse colon’ (TC), ‘descending colon’ (DC), ‘sigmoid and rectum’ (S&R). Fig 1 shows a typical scan in which increased ^18^FDG localisation (increased activity) in the DC segment (represented by the dark areas) can be clearly seen.

A subset of 10 scans was then randomly selected to perform an initial quantitative analysis of activity localised in the colon for each of the four segments. The summed pixel values for each segment were determined as a measure of the total activity, and hence total amount of ^18^FDG, in each segment. On inspecting these data, one patient was noted to have high activity in the sigmoid which was double that of any other segment studied. However, since non-parametric statistics were to be used, this subject was not excluded from analysis. The fractional activity in each segment, expressed as a percentage of the total activity in the colon, was determined. Segment lengths can vary between subjects. To investigate the possibility that differences in segment length gave rise to differences in the activity between segments, the activity/unit length was also determined. Once again, this was expressed as a percentage of the total activity in the colon.

The summed scores across the five observers from the qualitative data were subjected to a Friedman non-parametric 2-way ANOVA analysis utilising a χ^2^ test to investigate whether the observation of localisation of ^18^FDG in the colon was different between different segments [12,13]. A Wilcoxon matched-pairs signed-rank test was then used to investigate differences between pairs of segments [13]. Statistical significance was taken as p < 0.05 but for the Wilcoxon matched-pairs test a Bonferroni correction for multiple comparisons was applied, so p < 0.0083 was required for statistical significance at this level [12]. This same non-parametric ANOVA analysis was applied to both quantitative measures (the segmental activity and the activity/unit length) to determine whether these were different in different colonic segments.

## Results

Only 2 of the 30 patients had a median score ≤1 across all segments of the colon. In only one patient did a single observer conclude an absence of activity in all four segments.

**Fig 2:**
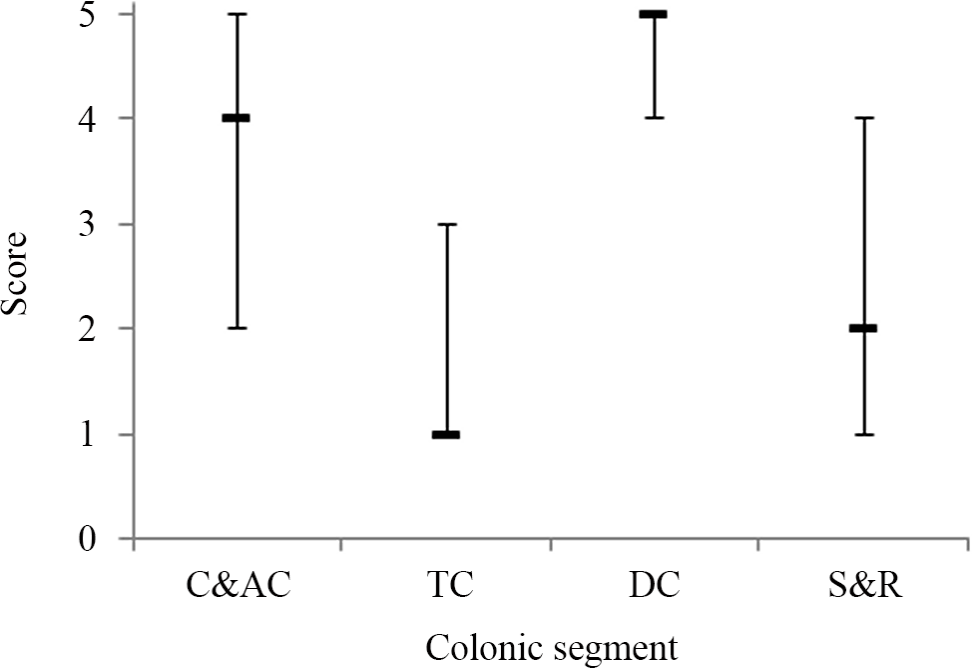
The 25^th^ centile, median, 75^th^ centile of the scores from 5 observers for the presence of ^18^FDG in the 4 sections of the colon

The median scores for each of the segments of the colon studied calculated across all observers are given in Table 1 and the median and interquartile range plotted in Fig 2.

**Table 1:** The median and mean±SD scores across all observers for the activity in different segments of the colon.

	C&AC	TC	DC	S&R
Median Score	4	1	5	2
Mean±SD	3.2±1.8	1.7±1.4	4.2±1.1	2.2±1.7

The non-parametric 2-way ANOVA showed a significant difference in the amount of ^18^FDG in the different segments (χ^2^ test, p < 0.001). The Wilcoxon matched-pairs test showed a statistically significant difference between the different segments for all segment pairs with the exception of the caecum and ascending colon and the descending colon (Table 2).

**Table 2:** Probabilities of a difference in ^18^FDG localisation between different segments of the colon for which the median values are given in Table 1. Statistically significant differences at p <0.05 (corresponding to p < 0.0083 with the Bonferroni correction) are shown in bold.

	C&AC	TC	DC	S&R
C&AC	-	**0.001**	0.011	**0.005**
TC		-	**<0.001**	**0.006**
DC			-	**<0.001**
S&R				-

**Fig 3:**
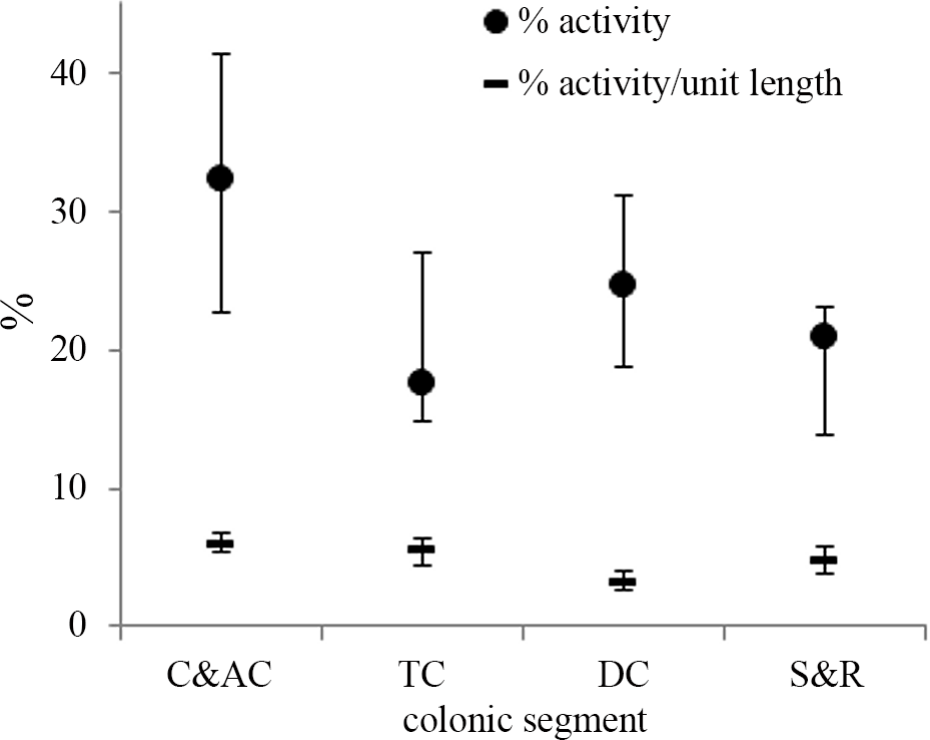
The 25^th^ centile, median, 75^th^ centile of the percentage activity (•) and percentage activity/unit length (˗) of ^18^FDG in the 4 sections of the colon

The mean and standard deviation for the percentage activity and the activity/unit length for each segment of the colon are given in Table 3. The large standard deviation in the sigmoid and rectum (S&R) is the result of one patient having a large percentage activity, as noted previously. Therefore Fig 3 shows the median and interquartile range for both measures.

**Table 3:** The mean±SD for the percentage activity and percentage activity/unit length for the different segments of the colon.

	C&AC	TC	DC	S&R
% activity	32±10	21±8	25±7	21±12
% activity/unit length	6.3±1.4	5.7±2.0	3.5±1.3	6.1±5.0

The non-parametric ANOVA analysis of the total activity in each segment showed no statistically significant difference (χ^2^test, p > 0.2). As expected, the Wilcoxon matched-pairs tests on the difference in activity between pairs of segments showed no statistically significant differences (Table 4).

**Table 4:** Probabilities of a difference in the total ^18^FDG activity between different segments of the colon for 10 subjects. The mean and standard deviation values are given in Table 3. Statistical significance at p < 0.05 requires p < 0.0083 with the Bonferroni correction.

	C&AC	TC	DC	S&R
C&AC	-	0.041	0.041	0.061
TC		-	0.148	0.327
DC			-	0.148
S&R				-

The non-parametric ANOVA analysis of the activity/unit length showed a statistically significant difference between segments (χ^2^ test, p < 0.01). It was noted that the descending colon had the lowest activity/unit length compared with the other three segments; all of which had very similar values (Fig 3). Wilcoxon matched-pairs tests showed the only statistically significant difference was between the caecum and ascending colon and the descending colon (Table 5).

**Table 5:** Probabilities of a difference in the ^18^FDG activity/unit length between different segments of the colon for 10 subjects. The mean and standard deviation values are given in Table 3. Statistically significant differences at p <0.05 (corresponding to p < 0.0083 with the Bonferroni correction) are shown in bold.

	C&AC	TC	DC	S&R
C&AC	-	0.441	**0.003**	0.107
TC		-	0.010	0.441
DC			-	0.013
S&R				-

## Discussion

We found that only 2 of the 30 patients studied had a median score ≤1 across all the colonic segments and across all observers. In only one patient did a single observer assign no activity to all four colonic segments. From these results we infer that localisation of ^18^FDG in the colon is not a chance finding but rather the norm in patients without colonic pathology that could account for cellular uptake of ^18^FDG (e.g. inflammatory disease or malignancy). Lack of any statistically significant differences from the analysis of the quantitative data, save for only one instance in the activity/unit length, shows there are only minimal differences in the average localisation of ^18^FDG across the colon. This is consistent with our findings of differences between colonic segments in the analysis of the qualitative data where the eye is drawn to focal localisation of the isotope and structure outlines (Fig 1)

Our results clearly show that localisation of ^18^FDG in the colon is a normal finding in subjects without colon related disease. Its presence, as noted in the introduction, is counter-intuitive since all sugar transport should have been completed in the small bowel. Therefore identifying ^18^FDG distribution and position within the colonic wall / lumen is important in understanding possible transport pathways. The presence of ^18^FDG in the colon could be explained by adherence to microbes [14] or food residue, or to it being present within the wall of the colon, or any combination of these. Any presence within the colonic wall includes presence in one or more of the mucosa, submucosa or muscle. However, we cannot differentiate further as at its minimum, the thickness of the colonic wall can be less than 10mm [4] whereas the spatial resolution of the PET scan is about 4mm. Nevertheless, there is persuasive evidence of the existence of ^18^FDG within the colonic lumen, as evidenced by its movement following a bowel stimulus comprising a high fat meal and coffee [10]. These findings provide evidence that ^18^FDG is present in the colonic lumen but these do not negate the possibility that it is simultaneously present in the colonic wall.

For ^18^FDG given by intravenous injection to reach the colonic lumen, it must either have crossed the colonic mucosa from the blood or have been delivered directly to the colon from the small intestine.

There is evidence of physiologic accumulation of ^18^FDG in the gall bladder [15]. This, by inference, would signify its delivery into the duodenum when the gall bladder contracts. PET imaging typically starts one hour after the injection of ^18^FDG, and all images are captured over the next 30 minutes. Small intestinal transit time is typically longer than 1 hour, and colonic transit time longer still, at between 6 and 24 hours. Therefore any ^18^FDG delivered to the colon from the small intestine will have just reached the caecum within our imaging protocol. The results from analysis of our qualitative data, however, show the highest localisation of ^18^FDG in the descending colon, whilst the analysis of the quantitative data indicates similar activity in all segments. Therefore our results do not support transfer of ^18^FDG from the duodenum as a likely pathway.

Interestingly, indirect support for the alternative pathway where ^18^FDG is delivered from the blood comes from observations made in the rare instances when ^18^FDG has had to be given orally because of poor venous access [16]. Images acquired 40 minutes after drinking the isotope show activity in the brain, heart, liver and bladder. This can only be explained by rapid absorption of the isotope into the blood pool. Transport of glucose from the small intestinal lumen to the blood pool is through SGLT1, which is specific for this molecule. All other glucoses, including ^18^FDG, are transported through the GLUT transporters on the cell apical surface.

Evidence for blood to gut lumen transfer in the small intestine comes from the animal work of Levine et. al., [17] which showed a good linear correlation between the glucose transport to the lumen of the small intestine and the blood glucose concentration which they conclude supports diffusion as the transport mechanism. We have anecdotal evidence that such a transport also may also exist in humans since ^18^FDG was found in the ileostomy bag of one of the patients considered for, but rejected from, inclusion in our study: lacking a colon the ^18^FDG could *only* have come from the blood, across the small intestinal wall and into the lumen.

Our view is that a similar mechanism exists in the colon where ^18^FDG is transported from the blood to the colonic lumen, a transport driven by concentration differences with higher levels in the blood directing flow down the gradient to the lumen. The colon is well perfused throughout its length. We found ^18^FDG in all segments of the colon with minimal difference in the quantified activity between them. This can be explained by a two-compartment model, where the compartments are separated by the colonic wall with the passage of material between compartments determined by the concentration difference between them. Such a pathway is, by its very nature, bi-directional but in the colon the luminal glucose concentration will be much lower than the blood concentration.

We further postulate that to reach the colonic lumen, ^18^FDG is more likely to travel through the para-cellular route, as that would probably offer less impediment than the trans-cellular route suggested by Wang et. al., [10]. Wang et. al., [10] postulated that the passage of ^18^FDG to the colonic lumen is through the GLUT transporters located on the basolateral surface of the mucosal cells. Such transporters are known to “export” various sugars and glucose analogues from mucosal cells to the blood in the small intestine but we have some reservation whether these transporters also exist in the colon and if they do exist whether they can also transport in the opposite direction. In addition, Wang et. al., [10] offer no explanation as to how any intra-cellular ^18^FDG is then transported from the apical surface into the colonic lumen.

One of the primary functions of the colon is to remove water from the digested food to create solid stool for excretion. If our hypothesis that ^18^FDG is transported across the wall of the colon by diffusion is proven correct, then ^18^FDG may provide a non-invasive probe into the functioning of the colon.

Other workers have reported “higher” ^18^FDG activity in the colon of patients treated with metformin for Type 2 diabetes [18]. We specifically wanted to look at subjects with normal colon function and a retrospective analysis showed that none of the subjects in our study were treated with metformin. Type 2 diabetes is characterised by “insulin resistance” with the hormone secreted in excess but where the cellular response is reduced, thus reducing its effectiveness and mimicking deficiency. Metformin, when used in treating diabetes with other anti-diabetic drugs, acts by optimising the effect of endogenous insulin. The result is decreased gluconeogenesis and increased peripheral utilisation of glucose. Any reduction of blood glucose *(see below)* would alter the fraction of circulating ^18^FDG; specifically any reduction in the circulating glucose would result in a proportionate increase in the fraction of ^18^FDG. Despite the role of metformin in the management of type 11 diabetes, the actual reduction in circulating glucose is, counterintuitively, small. We therefore suggest metformin “highlights” the movement of ^18^FDG across the colonic wall as the colon is well perfused throughout its length. Since metformin is the only licensed biguanide in clinical use, comparisons with similar drugs are not possible. Thus, unwittingly we may have identified a glucose/glucose-analogue pathway, which, is sensitive to the effects of insulin resistance and reduction.

## References

[1] Miraldi F, Vesselle H, Faulhaber P, Adler LP, Leisure GP. Elimination of artifactual accumulation of FDG in PET imaging or colorectal cancer. Clin. Nucl. Med. 1998;23(1):3–7 9442955 10.1097/00003072-199801000-00002

[2] Kim S-K, Chung J-K, Kim BT, Kim SJ, Jeong JM, Lee DS, et al Relationship between gastrointestinal F-18-fluorodexoyglucose accumulation and gastrointestinal symptoms in whole-body PET. Clin. Posit. Imaging 1999 2(5):273–279 10.1016/s1095-0397(99)00030-8 14516651

[3] Corman ML, Nicholls RJ, Fazio VW, Bergamaschi R. Corman’s Colon and Rectal Surgery. Philadelphia:Lippincott Williams and Wilkins, 2012

[4] Fisher J. Normal colon wall thickness on CT. Radiology 1982;145:415–418 7134445 10.1148/radiology.145.2.7134445

[5] Haber HP, Benda N, Fitzke G, Lang A, Langenberg M, Riethmüller J, et al Colonic wall thickness measured by ultrasound: striking differences in patients with cystic fibrosis versus healthy controls. Gut 1997;40:406–411 9135533 10.1136/gut.40.3.406 PMC1027094

[6] Wiesner W, Mortele KJ, Ji H, Ros PR. Normal colonic wall thickness at CT and its relation to colonic distension. J Comput Assist Tomogr 2002;26(1):102–6 11801911 10.1097/00004728-200201000-00015

[7] Dialer I, Hundt C, Bertele-Harms R-M, Harms HK. Sonographic evaluation of bowel wall thickness in patients with cystic fibrosis. J Clin Gastroenterol 2003;37(1):55–60 12811210 10.1097/00004836-200307000-00014

[8] Nordin N, Ab Rahma NS, Myint YM, Aminudin M, Abduljabbar HN, Pahl C, et al Wall thickness measurement of colon based on ultrasound image segmentation In: Pavelkova D, Strouhal J, Pasekova M, editors:. Advances in Environment, Biotechnology and Biomedicine. Athens:WSEAS LLC, 2012.

[9] Ozguven MA, Karacalioglu AO, Ince S and Emer MO. Altered biodistribution of FDG in patients with type-2 diabetes mellitus. Ann. Nucl. Med. 2014; 28(6);505–511. doi: 10.1007/s12149-014-0840-y 24652347 10.1007/s12149-014-0840-y

[10] Wang K, Chen YC, Palmer MR, Tal I, Ahmed A, Moss AC, et al Focal physiological fluorodeoxyglucose activity in the gastrointestinal tract is located within the colonic lumen. Nucl Med Commun 2012;33(6):641–647 doi: 10.1097/MNM.0b013e328350859b 22240934 10.1097/MNM.0b013e328350859b

[11] Otsuka H, Graham MM, Kubo A, Nishitani H. The effect of oral contrast on large bowel activity in FDG-PET/CT. Annals of Nuclear Medicine 2005;19(2):101–108. 15909489 10.1007/BF03027388

[12] Thomas JR, Nelson JK. Research Methods in Physical Activity. Illinois: Human Kinetic Books, 1990.

[13] Siegel. S. Nonparametric statistics for the behavioural sciences New York: McGraw Hill, 1956.

[14] Arasaradnam RP, Penny R, Cullis J, Williams N, Wilson A, Bardhan KD. 18F-Fluorodeoxyglucose: the Trojan horse of PET/CT. Nucl Med Commun. 2012;33(12):1312 23014244 10.1097/MNM.0b013e328359d3a6

[15] Murata Y, Watanabe W, Kubota K, Toda K, Nakamura S, Okouchi K, et al PET/CT evaluation of the physiologic accumulation of 18F-FDG within the gallbladder vesicle. Nuc. Med. Biol. 2007;34(8):961–966. 10.1016/j.nucmedbio.2007.07.006 17998099

[16] Franc B, Carlisle MR, Segall G. Oral administration of F-18 FDG to evaluate a single pulmonary nodule by positron emission tomography in a patient with intravenous access. Clin. Nuc. Med. 2003;28(7)541–544 2003 10.1097/00003072-200307000-00001 12819404

[17] Levine GM, Shiau Y-F, Deren JA. Characteristics of intestinal glucose secretion in normal and diabetic rats. Am. J. Physiol. 1982;242(5):G455–G459 6211101 10.1152/ajpgi.1982.242.5.G455

[18] Gontier E, Fourme E, Wartski E, Blondet C, Bonardel M, Le Stanc E, et al High and typical ^18^F-FDG bowel uptake in patients treated with metformin. Eur J Nucl Med Mol Imaging 2008;35:95–99 17786437 10.1007/s00259-007-0563-6

